# Prospective validation of a biomarker-driven response prediction model to romiplostim in lower-risk myelodysplastic neoplasms – results of the EUROPE trial by EMSCO

**DOI:** 10.1038/s41375-022-01669-z

**Published:** 2022-09-07

**Authors:** Anne Sophie Kubasch, Aristoteles Giagounidis, Georgia Metzgeroth, Anna Jonasova, Regina Herbst, Jose Miguel Torregrosa Diaz, Benoit De Renzis, Katharina S. Götze, Marie-Luise Huetter-Kroenke, Marie-Pierre Gourin, Borhane Slama, Sophie Dimicoli-Salazar, Pascale Cony-Makhoul, Kamel Laribi, Sophie Park, Katja Jersemann, Dorothea Schipp, Klaus H. Metzeler, Oliver Tiebel, Katja Sockel, Silke Gloaguen, Anna Mies, Fatiha Chermat, Christian Thiede, Rosa Sapena, Richard F. Schlenk, Pierre Fenaux, Uwe Platzbecker, Lionel Adès

**Affiliations:** 1 Department of Hematology, Cellular Therapy and Hemostaseology, Leipzig University Hospital, Leipzig, Germany; 2 German MDS Study Group (D-MDS), Leipzig, Germany; 3 The European Myelodysplastic Neoplasms Cooperative Group (EMSCO), Leipzig, Germany; 4 Department of Oncology, Hematology and Palliative Care, Marien Hospital, Düsseldorf, Germany; 5 Department of Hematology and Oncology, University Medical Centre, Mannheim, Germany; 6 1st Medical Department - Hematology, General Hospital, Prague, Czech Republic; 7 Medizinische Klinik III, Klinikum Chemnitz, Chemnitz, Germany; 8 Department of Hematology and Oncology, CHU de Poitiers, Poitiers, France; 9 Service d’Hématologie Clinique Adulte, Clermont Ferrand, France; 10 Department of Medicine III, Technical University of Munich, Munich, Germany; 11 Department of Hematology, Oncology, and Tumor Immunology, Charité-Universitätsmedizin Berlin, Berlin, Germany; 12 CHU Limoges, Limoges, France; 13 Service d’Hématologie, Centre Hospitalier d’Avignon, Avignon, France; 14 University Hospital Bordeaux, Pessac, France; 15 Centre Hospitalier Annecy-Genevois, Pringy, France; 16 Centre Hospitalier Du Mans, Le Mans, France; 17 Department of Hematology, CHU Grenoble, Grenoble, France; 18 GWT-TUD GmbH, Dresden, Germany; 19 DS-Statistics, Rosenthal-Bielatal, Germany; 20 Institute of Clinical Chemistry and Laboratory Medicine, Medical Faculty, Technical University Dresden, Dresden, Germany; 21 Department of Internal Medicine I, University Hospital Carl Gustav Carus, Technical University Dresden, Dresden, Germany; 22 Groupe Francophone des Myélodysplasies, Paris, France; 23 Department of Internal Medicine V, University Hospital of Heidelberg, Heidelberg, Germany; 24 NCT-clinical Trials Office, German Cancer Research Center, Heidelberg, Germany; 25 Hématologie Clinique, Hôpital Saint-Louis, Paris, France

**Keywords:** Haematological diseases, Translational research

## Abstract

The EUROPE phase 2 trial investigated the predictive value of biomarkers on the clinical efficacy of single agent romiplostim (ROM) treatment in patients with lower-risk myelodysplastic neoplasms (LR-MDS) and thrombocytopenia within the ‘European Myelodysplastic Neoplasms Cooperative Group‘ (EMSCO) network. A total of 77 patients with LR-MDS and a median platelet count of 25/nl were included, all patients received ROM at a starting dose of 750 μg by SC injection weekly. Thirty-two patients (42%) achieved a hematologic improvement of platelets (HI-P) with a median duration of 340 days. Neutrophil (HI-N) and erythroid (HI-E) responses were observed in three (4%) and seven (9%) patients, respectively. We could not confirm previous reports that HI-P correlated with baseline endogenous thrombopoietin levels and platelet transfusion history, but *SRSF2* mutation status and hemoglobin levels at baseline were significantly linked to HI-P. Sequential analysis of variant allelic frequency of mutations like *SRSF2* did not reveal an impact of ROM on clonal evolution in both responders and non-responders. In summary, our study confirms the safety and efficacy of ROM in LR-MDS patients and may allow to better define subgroups of patients with a high likelihood of response.

## Introduction

Ineffective hematopoiesis and peripheral cytopenia are the hallmark features of myelodysplastic neoplasms (MDS) [1, 2]. Patients with this clonal myeloid disorder carry a heterogeneous prognosis due to a highly variable risk of progression to acute myeloid leukemia (AML) [3] but also of mortality related to complications of cytopenia [4]. Until now, the “Revised International Prognostic Scoring System” (IPSS-R) [5] enables individual risk classification into lower (LR) vs. higher-risk (HR) MDS based on platelet counts and other clinical parameters. Around half of MDS patients present at diagnosis with thrombocytopenia [4, 6, 7], which is not only associated with a shortened survival due to increased bleeding risk, but also with a higher risk of progression to AML [8–14].

Romiplostim (ROM) and Eltrombopag (EPAG) are thrombopoietin receptor agonists (TPO-RA) [15–17], which both have already demonstrated clinical efficacy in treating MDS patients with thrombocytopenia [18–22]. Both ROM and EPAG stimulate megakaryocyte differentiation and proliferation [23, 24]. While the oral small molecule EPAG binds to a transmembrane site of the TPO-receptor [17, 25, 26] the peptide mimetic ROM binds directly at the extracellular TPO-receptor binding site where also native TPO binds [27–30]. Until now, both TPO-RA are not approved for the treatment of MDS patients with thrombocytopenia, thus regular platelet transfusions still represent the only supportive treatment option for these patients [16, 17]. ROM has shown safety and clinical efficacy in prospective randomized trials in LR-MDS [4, 12, 20, 31, 32]. The registration program was, however, stopped due to initial safety concerns with regards to disease progression potentially accelerated by ROM [20]. Subsequent retrospective analyses did not confirm this observation [20] but showed that lower baseline endogenous thrombopoietin (TPO) levels (<500 pg/ml) and limited platelet transfusion events (PTE) (<6 platelet units transfused in the past year) predicted a greater likelihood of a subsequent platelet response to ROM [33]. The EUROPE study was designed to prospectively validate these findings and to investigate additional biomarkers of response and safety in this large European phase 2 trial.

## Methods

### Eligibility criteria and patient disposition

The prospective EUROPE multicenter phase 2 trial (NCT02335268) within the “European Myelodysplastic Neoplasms Cooperative Group” (EMSCO) network investigated the predictive value of endogenous TPO levels and PTE as well as other biomarkers including molecular signature on the clinical efficacy of single agent ROM treatment. Patients older than 17 years and IPSS low or intermediate 1 risk groups were eligible after confirmation of MDS diagnosis by central morphology with a bone marrow blast count <5% and the mean of two measured platelet counts, not influenced by transfusions, ≤30/nl or ≤50/nl (in case of a bleeding history). Patients with a prior history of hematopoietic stem cell transplantation, HR-MDS, AML, aplastic anemia or other non-MDS related bone marrow stem cell disorders as well as after previous treatment with any other thrombopoietic growth factor, were not eligible for study inclusion. Moreover, patients with a history of arterial thrombosis within the past year or venous thromboses that currently require anti-coagulation therapy were excluded. All eligible patients underwent screening, previous transfusion history and endogenous TPO-levels were measured centrally. According to a previously published model of response to ROM [33], patients were assigned into two different cohorts at the time of screening based on their previous PTE and centrally assessed TPO serum levels (cohort A: TPO < 500 ng/l and PTE < 6 units/past year; cohort B: TPO > 500 ng/l, and/or PTE ≥ 6 units/past year).

### Trial design and end-point measures

The primary efficacy endpoint was the rate of hematologic improvement of platelets (HI-P) according to IWG 2006 criteria [34] defined as an absolute increase of platelets to ≥30/nl for patients starting at >20/nl or an increase of platelets from <20/nl to >20/nl and by at least 100 percent lasting for eight weeks or longer after at least 16 weeks of ROM treatment [34].

Secondary endpoints were rate of hematologic improvement of erythrocytes (HI-E), defined as increase of hemoglobin value by ≥1.5 g/dL or transfusion burden reduction by at least four RBC units over eight weeks [34], or neutrophils (HI-N), defined as for pretreatment absolute neutrophil count less than 1.0/nl an at least 100 percent increase as well as an absolute increase of more than 0.5/nl according to IWG 2006 criteria [34]. Additional secondary endpoints were incidence of disease progression to HR-MDS or AML, increase of peripheral blasts during therapy, bleeding events and the association of the presence of certain mutations with the individual disease course and response.

**Fig. 1:**
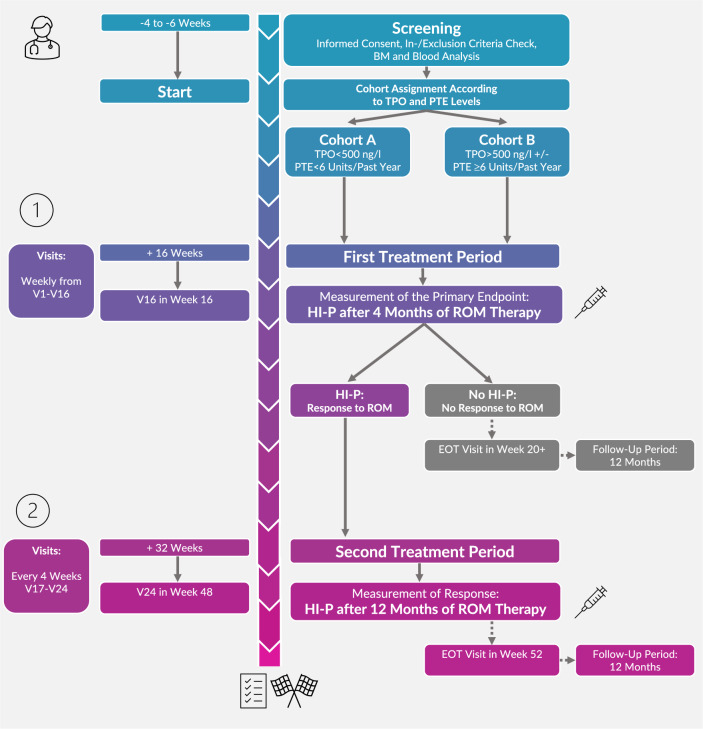
Study design of the EUROPE trial. After screening and eligibility check, patients were assigned into two different cohorts depending on their endogenous TPO-level and previous platelet transfusion events (PTE).

Patients were treated with ROM for a period of at least four months. If patients responded (HI-P), treatment was maintained for a total of up to one year duration (Fig. 1) and non-responding patients terminated treatment after the first four months. Included patients were monitored for survival, disease progression and bleeding events for additional 12 months following the end of study. With the aim to investigate the molecular signature of included patients, we applied a next-generation sequencing panel (TruSight Myeloid Sequencing Panel by Illumina which screened 54 myeloid candidate genes) (Table 1) at baseline and at the primary endpoint.

**Table 1:** Frequencies of somatic mutations at baseline.

Type of somatic mutation		Frequency			
		Non HI-P (*N* = 45)		HI-P (*N* = 32)	
Visit	Mutation	*N*	%	*N*	%
Screening	≥1 mutation	35	77.8	25	78.1
	≥2 mutations	22	48.9	18	56.3
	no mutations	3	6.7	2	6.3
	ABL1	0	0.0	1	3.1
	ASXL1	7	15.6	2	6.3
	BCOR	2	4.4	1	3.1
	BRAF	0	0.0	1	3.1
	CBL	2	4.4	1	3.1
	CBLB	0	0.0	1	3.1
	CEBPA	0	0.0	2	6.3
	CUX1	1	2.2	1	3.1
	DNMT3A	9	20.0	4	12.5
	EZH2	5	11.1	2	6.3
	FLT3	0	0.0	1	3.1
	GNAS	2	4.4	0	0.0
	IDH1	1	2.2	0	0.0
	IDH2	1	2.2	0	0.0
	KMT2A	0	0.0	2	6.3
	KRAS	1	2.2	1	3.1
	MPL	1	2.2	0	0.0
	MYC	1	2.2	0	0.0
	NF1	1	2.2	1	3.1
	NPM1	1	2.2	1	3.1
	NOTCH1	2	4.4	1	3.1
	NRAS	2	4.4	1	3.1
	PHF6	1	2.2	1	3.1
	PPM1D	0	0.0	1	3.1
	RAD21	0	0.0	1	3.1
	RUNX1	7	15.6	7	21.9
	SETBP1	0	0.0	1	3.1
	SF3B1	1	2.2	2	6.3
	SH2B3	1	2.2	0	0.0
	SMC3	1	2.2	0	0.0
	SRSF2	7	15.6	13	40.6
	STAG2	3	6.7	0	0.0
	TET2	11	24.4	10	31.3
	TP53	2	4.4	4	12.5
	U2AF1	6	13.3	3	9.4
	U2AF2	1	2.2	0	0.0
	WT1	1	2.2	0	0.0
	ZRSR2	1	2.2	2	6.3

ROM was initiated at a dose of 750 μg weekly by subcutaneous injection. During study, the dose was adjusted based on the patient’s platelet counts. The dose of ROM was withheld if the platelet count was ≥400/nl and then reinitiated at a reduced dose (adapted to 500 μg, 250 μg and 125 μg, respectively) at the next scheduled visit if platelet count was back to <400/nl. If platelet count was ≤50/nl before the next planned ROM application, dose was increased to the next higher dosing level until a maximum dose of 750 μg. In case of a central morphology confirmed persisting, and more than four weeks lasting, appearance of peripheral blasts between three to nine percent or in case of an increase in peripheral blasts to more than nine percent at any time point, ROM treatment was stopped. If peripheral blast count decreased to less than three percent during a four week “wash out” period, ROM treatment was reinitiated at the next scheduled visit. In patients with persisting peripheral blast counts greater than two percent after the “wash out” period, a bone marrow assessment was performed. In case of increasing bone marrow blasts to more than nine percent, the patient was withdrawn from the EUROPE study and a repeated bone marrow assessment was performed four weeks later. If the bone marrow blast count was less than five percent, included patients continued treatment at the next scheduled visit. Patients with five to ten percent blasts in the bone marrow could continue ROM treatment, but bone marrow evaluation was repeated after twelve weeks to exclude further disease progression.

### Statistical analysis

The EUROPE study was designed to prospectively validate that baseline TPO levels and platelet transfusion events (PTE) can predict response to ROM. The model prediction was considered successful if the best model group (Cohort A) achieves a significant better HI-P response rate after four months of ROM treatment than the worse group (Cohort B) with a clinically relevant Δ of 10% points. For the comparison of cohorts a sample size of 75 was determined given a 5% level of significance, a power of 80%, an expected recruitment ratio of 2:1 (cohort A: cohort B) and a dropout rate of 10%. The model prediction was examined by z-test for proportions and tested against one-sided alternative.

In general, comparisons of proportions between groups were analyzed using Fisher’s exact test or Pearson’s Chi-square test. Baseline characteristics were compared between groups by t-Test or Wilcoxon test. Time-to-event outcomes were evaluated by Kaplan–Meier estimators and compared between groups by log rank tests. Logistic regression was applied to assess the effects of age, gender and the presence of certain mutations on platelet response, whereby age was dichotomized by using the median age as cutoff. By stepwise backward elimination the logistic regression model was reduced to significant factors.

To decide whether the model to predict HI-P and non-HI-P can be improved by a different choice of characteristics for model group development, the influence of baseline variables (age, disease duration, hemoglobin, neutrophil count, platelet count, TPO, sex, cytogenetics, WHO classification, number of prior platelet transfusion units, cytopenia) was examined by logistic regression. A CHAID-analysis [35] was performed on basis of the significant variables identified by the simple logistic regression models (detailed method description in supplementary materials). All hypothesis except the primary endpoint were tested against a two-sided alternative. None of the hypotheses referred to more than two groups, therefore an adjustment for multiple comparisons was not necessary.

## Results

### Baseline characteristics of the patients

From 2015 to 2019, a total of 77 patients were included at 29 trial sites in Germany, France and the Czech Republic. Ethical approval for the EUROPE study was obtained by the institutional review boards at each trial site, written informed consent was provided by all patients before study inclusion. Fifty-one patients were stratified into cohort A and 26 into cohort B (baseline characteristics in Table 2A). In the whole cohort, 6% of patients were classified as IPSS-R very low risk and 87% as low or intermediate risk. Seven patients (9%) had chronic myelomonocytic leukemia 0 (CMML 0), and sixty-one patients (79%) had either MDS with single lineage dysplasia (MDS-SLD) or multilineage dysplasia (MDS-MLD). Two patients (3%) had MDS with single lineage dysplasia and ring sideroblasts (MDS-RS-SLD) and three patients (4%) had MDS with multilineage dysplasia and ring sideroblasts (MDS-RS-MLD). Three patients (4%) had MDS- unclassifiable (MDS-U). In patients with CMML 0 (*n* = 7) the *SRSF2* mutation was significantly more frequent (*n* = 5; 71%) compared to *SRSF2* wildtype (*n* = 2; 29%) (*p* < 0.05, *z*-test).

**Table 2:** **A**. Patient characteristics at baseline in the EUROPE trial. **B**. Hematologic response to ROM in the EUROPE trial.

Variable	Total (*n* = 77) number (%); median [range]	Cohort A (*n* = 51) number (%); median [range]	Cohort B (*n* = 26) number (%); median [range]
**A.**			
Age (years)	74 [42–93]	74 [42–93]	73 [58–86]
Female	28 (36)	18 (35)	10 (39)
MDS subtype			
MDS-SLD	15 (19)	6 (12)	9 (35)
MDS-RS-SLD	2 (3)	1 (2)	1 (4)
MDS-MLD	46 (60)	33 (65)	13 (50)
MDS-RS-MLD	3 (4)	1 (2)	2 (8)
MDS-u	3 (4)	3 (6)	0 (0)
CMML 0	7 (9)	6 (12)	1 (4)
Unknown	1 (1)	1 (2)	0 (0)
IPSS-R risk			
Very low	5 (6)	5 (10)	0 (0)
Low	52 (68)	37 (73)	15 (58)
Intermediate	15 (19)	6 (12)	9 (35)
High	3 (4)	2 (4)	1 (4)
Missing	2 (3)	1 (2)	1 (4)
Platelet count (/nl)	25 [3–48]	30 [9–48]	18 [3–45]
Serum TPO level (pg/ml)	90 [10–2000]	78 [10–366]	2000 [12–2000]
Platelet transfusion dependency	30 (39)	9 (18)	21 (81)
PTE/ last 8 weeks	0 [0–23]	0 [0–5]	4.5 [0–23]
Median baseline hemoglobin (g/dL)	10.35 [5–18.5]	11.85 [5–18.5]	9.34 [6.1–13.6]
RBC transfusion dependency	29 (38)	13 (26)	16 (62)
Median blast count BM (%)	1.5 [0–4]	2.0 [0–4]	1.0 [0–4]
**B.**			
**Response Type** *(IWG 2006)*	**Total (*n*** = **77)** **number (%); median [range]**	**Cohort A (*n*** = **51)** **number (%); median [range]**	**Cohort B (*n*** = **26)** **number (%); median [range]**
HI-P	32 (42)	24 (47)	8 (31)
HI-E	7 (9)	1 (2)	6 (23)
HI-N	3 (4)	3 (6)	0 (0)
Median treatment duration (days)	145 [12–576]	146 [12–379]	126 [15–576]
Median duration of HI-P (days)	340 [53 to >365)]	351 [53 to >365]	315 [153 to >340]

*TPO* trombopoietin, *PTE* platelet transfusion events, *RBC* red blood cell, *HI-P* hematologic improvement in platelets, *HI-E* hematologic improvement in hemoglobin, *HI-N* hematologic improvement in neutrophils.

At baseline, median age and duration of disease were 74 years and 13 months, respectively, 36% of enrolled patients were female. Median platelet count, TPO level and bone marrow blast count at screening were 25/nl, 90 pg/ml and 1.5%, respectively (Table 2A). Thirty patients (39%) were initially transfusion dependent of platelets and 29 patients (38%) of red blood cells (RBC). Twenty-four patients (31%) had received one or more prior therapy lines including lenalidomide (*n* = 2), erythropoiesis-stimulating agents (*n* = 12), luspatercept (*n* = 1), azacitidine (*n* = 8) or immunosuppressive treatments (*n* = 7). Seventeen patients (33%) in cohort A discontinued the study before the primary endpoint evaluation due to adverse events (*n* = 5) including cholecystitis (*n* = 1), mucosal hemorrhage (*n* = 1), leukocytosis (*n* = 1), bone pain (*n* = 1) and pulmonary embolism (*n* = 1); disease progression (*n* = 2) to HR-MDS (*n* = 1) and AML (*n* = 1), investigator decision (*n* = 5) due to no response (*n* = 4) and because of duodenal ulcer bleeding (*n* = 1), withdrawal of consent (*n* = 3) and increase of peripheral blasts (*n* = 2). In cohort B, six patients (23%) discontinued study before week 16 due to an adverse event (*n* = 1, oropharyngeal cancer), death (*n* = 1), disease progression to AML (*n* = 1), investigator decision (*n* = 1, acute pancreatitis) and withdrawal of consent (*n* = 2).

### Primary end point - platelet response

In the entire cohort, the median increase in platelet count during ROM treatment was 47/nl (range −25/nl to 343/nl). The study did not meet the primary endpoint, thirty-two out of 77 (42%) patients responded (HI-P) with only a numerically higher response rate in cohort A (47%, *n* = 24) vs. cohort B (31%, *n* = 8) (*p* = 0.295, z-test) (Table 2B).

Patients responded to ROM across all included WHO subtypes [36] without significant differences. Out of the HI-P responders (*n* = 32), nineteen (60%) had normal karyotype, two patients (12%) each had del(11q) and del(20q), one patient (3%) had trisomy 8 (+8) and four patients (13%) had any other single or double cytogenetic abnormality. Two responders (6%) had been classified as IPSS-R very low risk, twenty-five (78%) as low- and five (16%) as intermediate risk at baseline. Ten of 32 responders (31%) were initially transfusion dependent for platelets and seven (70%) became transfusion independent. Nine (28%) responders were depended on RBCs and one (11%) became transfusion independent during ROM treatment.

Median time from diagnosis in responders was eight months compared to 13 months in non-responders (*p* = 0.895, Wilcoxon test). Median platelet count, hemoglobin level and absolute neutrophil count in responders compared to non-responders at baseline were 29/nl and 22/nl, 11.6 g/dl and 9.7 g/dl, 1.72/nl and 1.69/nl, respectively and did not show significant differences. In responders, median serum TPO levels at baseline was 86 pg/ml compared to 90 pg/ml in non-responders (*p* = 0.664, Wilcoxon test). Interestingly, median PTE (responders (R): 0, range 0–15; non-responders (NR):0, range 0–23) and median number of RBC transfusions (R: 0, range 0–15; NR: 0, range 0–20) were not significantly different comparing responders and non-responders at baseline. Moreover, also median peripheral (R: 1.5%, range 0–4%; NR: 1.5%, range 0–4%) and bone marrow blast count (R: 1%, range 1–3%; NR: 1%, range 0–3%) as well as number of somatic mutations were not significantly different comparing responders vs. non-responders at baseline (Table 1). Responders displayed an *SRSF2* mutation significantly more frequently (*n* = 13; 41%) at baseline compared to non-responders (*n* = 7; 16%) (*p* = 0.018, Fisher’s exact test).

In the entire cohort, median time to first peak increase in platelet count was 39 days (range, 13 to 378 days) and the median time to HI-P was 14 days (range, 6 to 77 days). Median duration of HI-P in the whole cohort was 340 days (range, 53 to >365 days) and was significantly longer for patients in cohort A (351 days, range 53 to >365 days) compared to cohort B (315 days, range 153 to >340 days)) (*p* = 0.006, log-rank-test).

### Other hematological response

At 16 weeks of ROM treatment, three (4%) and seven (9%) patients had additional neutrophil (HI-N) and erythroid (HI-E) responses, respectively. Median time to first peak increase in neutrophil- and hemoglobin level was 99 days (range, 50 to 272 days) and 217 days (range, 14 to 385), respectively. Median duration of HI-E in cohort A was 189 days (range 70 to >287 days) and could not be estimated in cohort B. Median duration of HI-N in cohort A was 62 days, no HI-N in cohort B was observed. Interestingly, at the end of the study the proportion of patients who achieved HI-E was significantly larger in cohort B (*n* = 12, (55%)) compared to cohort A (*n* = 1, (5%)) (*p* = 0.001, Fisher’s exact test). None of the patients achieved concomitant trilineage responses (HI-P, HI-E and HI-N).

### Safety

During the first four months of treatment, 27 patients (35%) consistently received the full ROM dose of 750 μg without need for dose reduction. In six patients (8%), dose was adapted to 500 µg and in one patient (1%) to 125 µg due to platelet count increase ≥400/nl. In 43 patients (56%), treatment was temporarily stopped and then reinitiated for multiple reasons (see section “trial design and end-point measures” for dose adaptation rules).

The most frequent possible treatment-related adverse events (TRAEs, *n* = number of events) were diarrhea and nausea (*n* = 23), arthralgia and myalgia (*n* = 19), headache and dizziness (*n* = 12), hypertension (*n* = 9), injection site hematoma (*n* = 9), chest pain (*n* = 6), fatigue (*n* = 5) and peripheral edema (*n* = 5). In ten patients (13%) including five responders and five non-responders, leukocytosis and/or monocytosis were reported (day of first occurrence: range 8 to 147), which was reversible after dose reduction and/or interruption. Moreover, two cases of deep vein thrombosis 12 and 24 days after first ROM administration were reported among non-responders as possibly TRAEs.

One cerebrovascular accident (CTCAE grade 3) in a responder 22 weeks after starting ROM, one pulmonary embolism (CTCAE grade 4) in a non-responder at seven weeks of ROM and one case of asthenia (CTCAE grade 3) were reported as serious adverse events CTCAE ≥ grade 3 possibly attributed to ROM. This is in line with literature reporting a slightly higher rate of thromboembolic events in patients during TPO-RA treatment compared to patients without TPO-RA [4, 16, 19, 37, 38].

During treatment, six patients (8%, three responders and three non-responders) had transient (range of first occurrence: 19 days to 6 months after ROM initiation) appearance of centrally assessed peripheral blasts to more than ten percent, which was reversible after ROM interruption and two patients (2.6%) progressed to AML after four and five weeks of ROM treatment, respectively. Thirteen patients (17%) had grade two or higher bleeding adverse events, which were attributed to underlying thrombocytopenia and not to ROM treatment. One patient died during study not related to ROM treatment. Among the 77 included patients, eight (10%) had disease progression (R, *n* = 4, NR, *n* = 4) until the end of study with the earliest progression after 15 days and the latest after 247 days. Among patients with disease progression during study, five patients had normal karyotype, and one patient each had deletion 20q (del(20q)), deletion 5q (del(5q) and trisomy 8 (+8) at baseline. A total of 39 patients continued to receive ROM in the extension phase of the study after the first four months of treatment.

### Analysis of predictors of platelet response

We saw no significant ten percent points difference in the rate of HI-P comparing cohort A vs. cohort B, thus our findings do not support that both variables (TPO/PTE) alone allow reliable prediction of response to ROM [33]. With the aim to identify other potential variables as predictors of treatment response we performed a univariate analysis including multiple baseline parameters, but neither age, sex, disease duration, hemoglobin level, neutrophil or platelet count, TPO level, cytogenetic abnormalities, WHO type, platelet or RBC transfusion dependency, peripheral or bone marrow blast count nor number of cytopenia or somatic mutations were predictive of response to ROM.

**Fig. 2:**
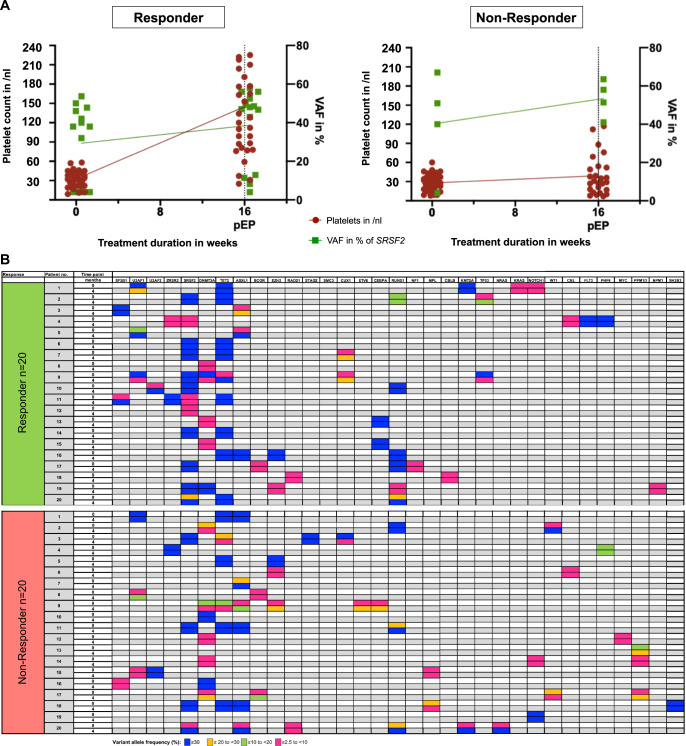
Mutational landscape of patients included in the EUROPE trial. **A** SRSF2 allelic burden and median platelet count in responders vs. non-responders. The course of median SRSF2 allelic burden and median platelet counts were compared at screening and after 16 weeks (primary endpoint, pEP) of ROM treatment. **B** Course of variant allelic burden in the EUROPE trial. Pattern of variant allelic burden at screening and after 16 weeks (pEP) of ROM treatment. No clinically relevant changes in clonal burden were observed in responders compared to non-responders.

Aiming to identify potential molecular patterns correlating with response, we analyzed patients for somatic variants in 54 myeloid candidate genes using a targeted next-generation sequencing panel at baseline and at week 16 (Table 1). At baseline, 72 of 77 patients (94%) were identified with somatic variants in genes recurrently mutated in myeloid malignancies. Most frequently mutated genes were *ASXL1* (R, *n* = 2 (6%), NR, *n* = 7 (16%)) *U2AF1* (R, *n* = 3 (9%), *NR*, *n* = 6 (13%)), *DNMT3A* (R, *n* = 4 (13%), *NR*, *n* = 9 (20%)), *RUNX1* (R, *n* = 7 (22%), *NR*, *n* = 7 (16%)), *SRSF2* (R, *n* = 13, (41%), *NR*, *n* = 7 (16%)), *EZH2* (R, *n* = 2 (6%), *NR*, *n* = 5 (11%)) and *TET2* (R, *n* = 10 (31%), *NR*, *n* = 11 (24%)). Moreover, we investigated the course of variant allele frequencies of clones identified at baseline during ROM treatment. We saw no clinically relevant changes of clonal size during therapy, neither in responders nor in non-responders, nor of any specific gene (Fig. 2A, B). Exemplarily, this is depicted by illustrating the course of the variant allele frequency of the *SRSF2* mutation in Fig. 2A. This observation indicates that ROM does not accelerate disease progression and thus underlines the safety of ROM in this therapeutic setting.

**Fig. 3:**
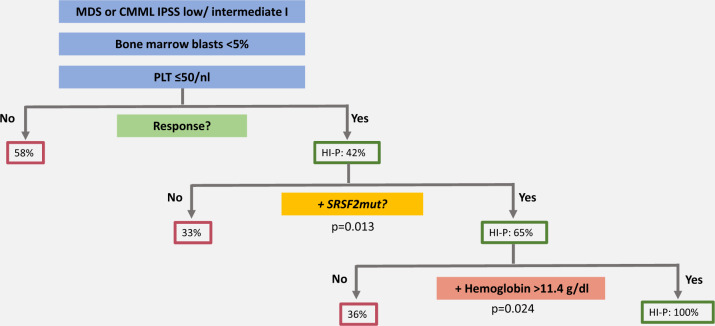
Response prediction model to Romiplostim based on the results of the EUROPE trial. The newly developed response prediction model contains the SRSF2 mutation status in combination with platelet count and hemoglobin level (threshold 11.4 g/dl).

We identified the presence of an *SRSF2* mutation as a significant predictor of response to ROM treatment (*p* = 0.016, logistic regression). Mutated *SRSF2* was significantly more frequent in responders (41%) compared to non-responders (16%) (*p* = 0.018, Fisher’s exact test). In patients with an *SRSF2* mutation, the probability to achieve HI-P was 65% compared to 33% in patients with *SRSF2* wildtype (Fig. 3). In non-responders, bone marrow hypercellularity was significantly more frequent (27%) compared to responding patients (0%) (*p* < 0.05, z-test), but we detected no significant differences comparing the rate of hypocellularity or the amount of megakaryocytes in the bone marrow in both groups.

### Development of a biomarker driven response prediction model

Because the previously published TPO/PTE based response predicting model to ROM [33] could not be validated in the EUROPE study, we finally developed an alternative response prediction model with the aim to improve personalized patient stratification in the future. The following variables at baseline were examined as possible predictors of platelet response by univariate logistic regression: ´age, gender, disease duration, hemoglobin-, platelet- and neutrophil count, serum TPO level, WHO subtypes, cytogenetic aberration, RBC and platelet transfusion dependency, number of cytopenias, number of mutations, peripheral/ bone marrow blast count and *DNMT3A, RUNX1, SRSF2, TET2, ASXL1, U2AF1* and *EZH2* mutation status. Only those variables, that were found to be significant at the ten percent level in the univariate regression were included in the multivariate CHAID-analysis [35] and only mutations which occurred in sufficiently large numbers in patients (≥7) were evaluated as possible predictors for HI-P. Thus, the CHAID-analysis incorporated hemoglobin-, platelet count, and different combinations of *DNMT3A, RUNX1, SRSF2, TET2, ASXL1, U2AF1* and *EZH2* as possible predictors of response. The percentage of correctly predicted HI-P was highest for the model, which included the platelet count, *SRSF2* mutation status and the hemoglobin level using the threshold of 11.4 g/dl and resulted in an overall accuracy of 70 % for a correct ROM response prediction (Fig. 3, Table 3). The threshold for the hemoglobin level was generated by the CHAID procedure and results from the final step of the merging process of predictor categories (for details see supplementary materials).

**Table 3:** Evaluation of the quality of model prediction.

HI-P at Week 16		Predicted Frequencies		Percentage correct	Cohen’s Kappa
		no	yes		
Observed Frequencies	**HI-P**	N	N	%	
	no	45	0	100.0	0.314
	yes	23	9	28.1	
**Total**	**%**	**88.3%**	**11.7%**	**70.1%**	

*HI-P* Hematologic improvement in platelets.

Our final model now predicts in LR-MDS patients with thrombocytopenia (platelet count ≤50/nl) an HI-P rate of 42% after ROM treatment, in patients additionally harboring an *SRSF2* mutation an HI-P rate of 65% and in *SRSF2mut* patients with baseline hemoglobin levels greater than 11.4 g/dl an HI-P rate of 100% compared to 36% in patients with hemoglobin levels lower than 11.4 g/dl (*p* = 0.024) (Fig. 3).

## Discussion and conclusion

The results of the EUROPE multicenter phase 2 trial confirm and strengthen reported data of earlier studies showing clinical efficacy of single agent ROM in a large subset of patients with LR-MDS. However, we could not validate the predictive value of baseline endogenous TPO levels and platelet transfusion history for consecutive platelet response. In fact, the study did not meet its primary endpoint (HI-P cohort A: 47%, cohort B: 31% (*p* = 0.295)). Therefore, both variables should not be used in clinical practice to select potential patients benefitting from this treatment.

The median duration of HI-P response of 340 days in the whole cohort is very encouraging and the safety profile in the EUROPE study was similar to previous studies investigating ROM in myeloid diseases with the most frequent treatment-related adverse events representing headache and dizziness, arthralgia, myalgia as well as gastrointestinal disturbances. We relied on a central morphology at inclusion and during monitoring of patients which we believe is important for the clinical use of ROM in these patients, to optimally monitor potential hematological side effects like leukocytosis and/or monocytosis seen in 13% of our patients. Although an earlier study [20] had highlighted the potential risk of ROM in accelerating development of AML or an increase of peripheral/ bone marrow blasts in MDS patients, our study confirms recently reported long-term follow-up data from a randomized controlled trial that showed no increased rate of leukemic progression during ROM treatment [4].

Clonal assessments during ROM treatment have not been published so far. We were therefore very interested not only to see whether potential molecular biomarkers predicted response to ROM, but also how these markers evolved during therapy. At first, we identified the presence of an *SRSF2* mutation as a significant predictor of response to ROM treatment (*p* = 0.016, logistic regression). In patients with an *SRSF2* mutation, the probability to achieve HI-P was 65% compared to 33% in patients with *SRSF2* wildtype. For responders as for non-responders, we did not find clinically relevant changes of variant allelic burden of mutations like *SRSF2* detected pre- and post-ROM, which underlines the safety of ROM in this therapeutic setting.

Comparing our here reported results with the recently described data investigating EPAG monotherapy in patients with LR-MDS [22], similar to our observations no differences in clonal size during EPAG therapy were reported [22]. In contrast to the EUROPE study which included only LR-MDS patients with thrombocytopenia, this phase 2 study included patients with any cytopenia (platelet count ≤30/nl or hemoglobin ≤9.0 g/dL or absolute neutrophil count ≤0.5/nl or platelet/RBC transfusion dependency) [22], six of 25 included patients (24%) achieved HI-P after 16–20 weeks of EPAG treatment [22]. Moreover, no disease progression to AML and no thromboembolic events were reported during EPAG treatment [22]. Thus, the clinical efficacy of EPAG in this therapeutic setting was also confirmed within this trial [22] suggesting that both TPO-RA have a comparable activity and safety in LR-MDS.

In conclusion, this prospective study did not confirm the general predictive value of TPO-levels and PTE on the response to ROM in patients with LR-MDS. Nevertheless, ROM is safe and highly efficacious in a large subset of patients. In the absence of approved treatment options for LR-MDS patients with thrombocytopenia, the application of TPO-RA will become even more important in the management of these patients with the high unmet need to reduce potentially fatal bleeding complications. To achieve this goal of a broader clinical use, further larger, prospective and controlled studies are warranted to specify the definitive role of TPO-RA in the treatment landscape of LR-MDS. Our newly developed response prediction model including *SRSF2* mutation status may help identify patients with the highest likelihood of response to ROM also in future clinical trials. To avoid overfitting of variables and to confirm our results, the here presented response prediction model needs to be validated in future studies.

## Supplementary information

Supplement material
