# Supplementary material for: Prospective validation of a biomarker-driven response prediction model to romiplostim in lower-risk myelodysplastic neoplasms – results of the EUROPE trial by EMSCO
Source: Leukemia. 2022 Sep 7;36(10):2519–27. doi: 10.1038/s41375-022-01669-z (PMC9522582; doi:10.1038/s41375-022-01669-z)
Supplement: Supplementary file 1 — Supplement material [file 41375_2022_1669_MOESM1_ESM.docx]

**Supplement materials**

*The CHAID method description is sourced directly from the following homepage: https://statisticasoftware.wordpress.com/2012/06/13/popular-decision-tree-chaid-analysis-automatic-interaction-detection/*

*Original quote STATISTICA:*

**“Method Description: CHAID Analysis**(1–4)

CHAID stands for “Chi-squared Automatic Interaction Detector” and is a tree classification method, the algorithm allows to construct (non-binary) trees relying on the Chi-square-test for categorical variables to determine the best next split at each step. For continuous dependent variables the algorithm uses the F-test(1–4).

The algorithm proceeds as follows:

**Preparing predictors.** The first step is to create categorical predictors out of any continuous predictors by dividing the respective continuous distributions into a number of categories with an approximately equal number of observations. For categorical predictors, the categories (classes) are “naturally” defined(1–4).

**Merging categories.** The next step is to cycle through the predictors to determine for each predictor the pair of (predictor) categories that is least significantly different with respect to the dependent variable; for classification problems (where the dependent variable is categorical as well), it will compute a *Chi*-square test (Pearson *Chi*-square); for regression problems (where the dependent variable is continuous), F tests. If the respective test for a given pair of predictor categories is not statistically significant as defined by an alpha-to-merge value, then it will merge the respective predictor categories and repeat this step (i.e., find the next pair of categories, which now may include previously merged categories). If the statistical significance for the respective pair of predictor categories is significant (less than the respective alpha-to-merge value), then (optionally) it will compute a Bonferroni adjusted *p*-value for the set of categories for the respective predictor(1–4).

**Selecting the split variable.** The next step is to choose the split variable, the predictor variable with the smallest adjusted *p*-value, i.e., the predictor variable that will yield the most significant split; if the smallest (Bonferroni) adjusted *p*-value for any predictor is greater than some alpha-to-split value, then no further splits will be performed, and the respective node is a terminal node(1–4).

The process is continued until no further splits can be performed (given the alpha-to-merge and alpha-to-split values).**”**

**References**

1. Kass G v. An Exploratory Technique for Investigating Large Quantities of Categorical Data. Applied Statistics. 1980;29(2):119.

2. Ripley BD. Pattern Recognition and Neural Networks. Cambridge University Press; 1996.

3. Morgan JN. THAID: A SEQUENTIAL ANALYSIS PROGRAM FOR THE ANALYSIS OF NOMINAL SCALE DEPENDENT VARIABLES. 1973.

4. STATISTICA. https://statisticasoftware.wordpress.com/2012/06/13/popular-decision-tree-chaid-analysis-automatic-interaction-detection/. 2012.
